# A Comparison of Gelling Agents for Stable, Surfactant-Free Oil-in-Water Emulsions

**DOI:** 10.3390/ma15186462

**Published:** 2022-09-17

**Authors:** Ji Yun Lee, Sang Ho Lee, Seon Ae Hwangbo, Tae Geol Lee

**Affiliations:** 1 Nano Safety Team, Safety Measurement Institute, Korea Research Institute of Standards and Science (KRISS), 267 Gajeong-ro, Yuseong-gu, Daejeon 34113, Korea; 2 Department of Chemical and Biomolecular Engineering, College of Engineering, Yonsei University, 50 Yonsei-ro, Seodaemun-gu, Seoul 03722, Korea; 3 LG Household & Health Care, Technical Process Research Team, 175 Gajeong-ro, Yuseong-gu, Daejeon 34114, Korea

**Keywords:** emulsion, surfactant-free, oil-in-water, gelling agent, emulsion stability

## Abstract

Emulsions have a range of applications, for example, in cosmetics, pharmaceuticals, and food. However, the surfactants used to prepare such emulsions can often be toxic to humans and the environment and also affect the oil properties of emulsions. Therefore, interest in surfactant-free emulsions has increased in recent years. One method to enhance emulsion stability without a surfactant is to use a gelling agent to increase the viscosity. Gelling agents are viscous hydrocolloids that gel when dispersed in water, even at low concentrations. In this study, we prepared six oil-in-water emulsions (oil content 20%) with different gelling agents (xanthan gum, Carbopol 981, TR-2, and Ultrez 20) and investigated the effect of the gelling agent concentration. For each sample, particle size and emulsion stability analysis were performed at high temperatures to ensure the stability of the emulsions. We observed that the emulsion prepared using TR-2 (0.25 wt%) did not aggregate at high temperatures for one month. Based on our assessment of the stability of these emulsions under various conditions, we believe that the use of gelling agents for the preparation of surfactant-free emulsions shows great promise for applications requiring long-term stable emulsions, such as cosmetics and medicine.

## 1. Introduction

Emulsions are a colloidal mixture of a liquid in another liquid; typically, the liquids are immiscible [1,2]. Generally, the following three conditions must be satisfied to form an emulsion: first, the two liquids must not mix with each other or must be mutually insoluble; second, sufficient stirring must be performed to disperse one liquid into another; finally, a surfactant or a combination of surfactants is necessary [3]. Water and oil are representative immiscible liquids, but the two can be mixed into oil-in-water (O/W) and water-in-oil (W/O) emulsions under the right conditions. Such emulsions have a range of uses in cosmetics, medicine, food, and paint. In particular, cosmetics and pharmaceuticals present a high absorption rate of functional substances, and for excellent product performance, require uniform and stable emulsions [4,5,6,7].

Thermodynamically, emulsions are unstable. In particular, lipids are hydrophobic and have high interfacial tension; thus, emulsions of water and oil without additives separate immediately after preparation. However, the addition of surfactants (emulsifiers), which typically have amphiphilic molecular structures, can be used to form stable emulsions. Surfactants typically have a hydrophilic head group and non-hydrophilic hydrocarbon chain(s) tails and can form micelle aggregates in aqueous solutions [8]. More than 20,000 surfactants have been identified to date, and these are widely used to prepare emulsions having a range of applications [9,10]. However, surfactants are readily absorbed into the human body and can remain in organs such as the heart, lungs, liver, and brain for up to five days. This accumulation has been linked to genetic changes, which can result in illness [9,10]. In addition, surfactants affect protein and biochemical components, and frequent exposure to surfactants can cause protein deformation and damage to the skin barrier. Further, in emulsions, the surfactant interacts with other emulsion components, potentially altering their function. Thus, the addition of surfactants not only reduces the purity of the emulsion, but also makes it difficult to study the role of the oil component on emulsion properties [11]. In addition, limitations in the development of new products arise due to limitations in the autonomy and functionality of product formulations. Thus, as a result of production costs, the time-consuming emulsion production process, and the potential for causing harm to the human body and the environment, the development of surfactant-free emulsification technology is essential and has recently drawn significant interest [12,13,14,15].

In this study, oil-in-water emulsions containing 20% oil were prepared without a surfactant. A gelling agent is added to increase viscosity, which is one of the characteristics involved in the stability of the emulsion. Gelling agents are hydrocolloids with a heterogeneous group of long-chain polymers (for example, polysaccharides and proteins) that form viscous mixtures or gels when dispersed in water, which can be exploited to modify the texture and viscosity of an emulsion [16]. A gel is formed through the formation of a three-dimensional network, such as the bonding or crosslinking of polymer chains, and then, the water in it is trapped or fixed to form a rigid structure that resists flow. The degree to which the viscosity is increased depends on the type and characteristics of the hydrocolloid; some show low viscosity at a fairly high concentration, but most show very high viscosity even at concentrations around 1% [17]. The gelling agent is concentrated by the non-specific entanglement of the three-dimensional disordered polymer chains. This is essentially a polymer-solvent interaction, and, below a critical concentration, the polymer dispersion shows Newtonian behavior, however, above the critical concentration, non-Newtonian behavior occurs [18]. In this study, we prepared surfactant-free emulsions by adding four types of gelling agents: xanthan gum, Carbopol 981, TR-2, and Ultrez 20, and investigated the effect of the concentration of the gelling agent on the stability of the emulsion, focusing on the changes in emulsion particle size and the stability at high temperatures and under harsh storage conditions.

## 2. Materials and Methods

### 2.1. Preparation of Oil-in-Water Emulsions

The emulsions were prepared from distilled water and grape seed oil (DAYDAYTHERAPY, Goyang, Korea). As gelling agents, xanthan gum (KELTROL F; CP Kelco, Atlanta, GA, USA), Carbopol 981 (LUBRIZOL, Wickliffe, OH, USA), Pemulen TR-2 (LUBRIZOL, Wickliffe, OH, USA), and Carbopol Ultrez 20 (LUBRIZOL, Wickliffe, OH, USA) were used. Tris Amino Ultra PC (Angus Chemical, Buffalo Grove, IL, USA) was used as a neutralizer, and the ratio of the gelling agent to the neutralizer was 1:1. In the prepared emulsion, the oil-to-water ratio was 20:80 (wt%). Different amounts of each gelling agent were added to yield emulsions having similar viscosities. A ROBOMICS (PRIMIX, Hyogo, Japan) mixer was used to stir the mixtures for emulsion production. In addition, a MAZERUSTAR KK-250S (KURABO, Osaka, Japan) was used as a defoaming machine to remove air bubbles in the emulsion after stirring. The processes used for emulsion production are listed in Table 1.

### 2.2. Emulsion Characterization

#### 2.2.1. Size Distribution of Emulsions Prepared with Gelling Agents

A laser particle size analyzer (LA-960; HORIBA, Kyoto, Japan) was used to measure the size of the oil particles in the emulsions containing different gelling agents, and the Mie scattering of light was used to calculate the particle size. Light sources with two different wavelengths were used: 650 and 405 nm, and the samples were diluted 100 times with distilled water before measurement. For size distribution analysis, it was performed in range of 86% to 85% of the transmittance of the blue light source (405 nm).

#### 2.2.2. Stability under High-Temperature

For high-temperature stability analysis, the emulsion was held at 45 °C, and particle size measurements were performed using the LA-960 device every week, for a month.

In addition, a Turbiscan (Leanontech) device was used to analyze the high-temperature emulsion stability. An 880 nm light source was used to evaluate the dispersion fundamentals of the emulsion. Measurements were carried out every 8 h for 30 days, at 45 °C, and the change in the delta backscattering of the emulsion was measured. The stability index, with respect to time from the bottom to the top of the sample, is denoted as TSI, as given by Equation (1). The mean value of TSI changes with the stability of the different emulsion particles [19]. This index is given by the following Equation (1):

(1) $$TSI=\sum_{i}\frac{\sum_{h}\left|scan_{i}\left(h\right)-scan_{i-1}\left(h\right)\right|}{H}$$

Here, *TSI* is the Turbiscan stability index, *H* is the sample height from the bottom of the cell to the meniscus, *scan_i_(h)* is the *_i_(h*) scan at a given height *h*, *scan_i−_*_1_*(h)* is the *_i−_*_1_*(h)* scan at a given height h, and i is the index from 1 to *k* (*k* = total time/scan speed).

In this study, the *TSI* (global) of the total sample height from the bottom of the cell to the meniscus is reported.

#### 2.2.3. Stability under Harsh Storage Conditions

If emulsions are commercially distributed, it is highly likely that they will be exposed to a range of environments such as very low or high temperatures during transportation. Therefore, the stabilities of the emulsions under harsh conditions were assessed.

Specifically, each sample was subjected to five 24 h cycles in the order of room temperature (25 °C), −10 °C, room temperature again, and high temperature (45 °C). The stability change of the emulsions is observed at each cycle.

## 3. Results and Discussion

### 3.1. Particle Size Distribution of Emulsions Prepared with Different Gelling Agents

**Figure 1:**
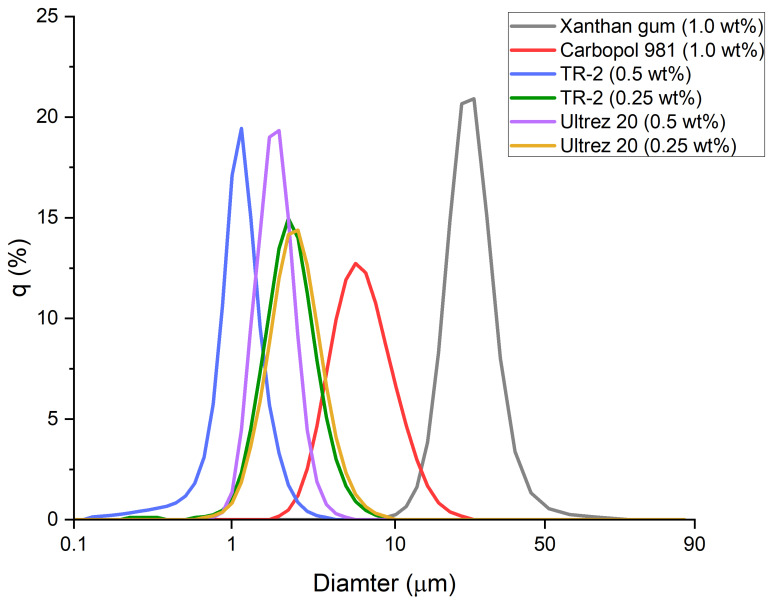
Particle size distribution of emulsions prepared with different gelling agents.

Immediately after the O/W (20%) emulsions had been prepared, the particle size distributions were measured (Figure 1). The emulsion prepared using xanthan gum contained the largest particles, which measured tens of micrometers in size. In contrast, the other gelling agents yielded particles having diameters of several micrometers. In particular, the emulsions prepared using 0.5 and 0.25 wt% of Ultrez 20 or TR-2 produced smaller and more uniform particles when 0.5 wt% gelling agent was used.

### 3.2. High-Temperature Stability

#### 3.2.1. Particle Size Distribution

**Figure 2:**
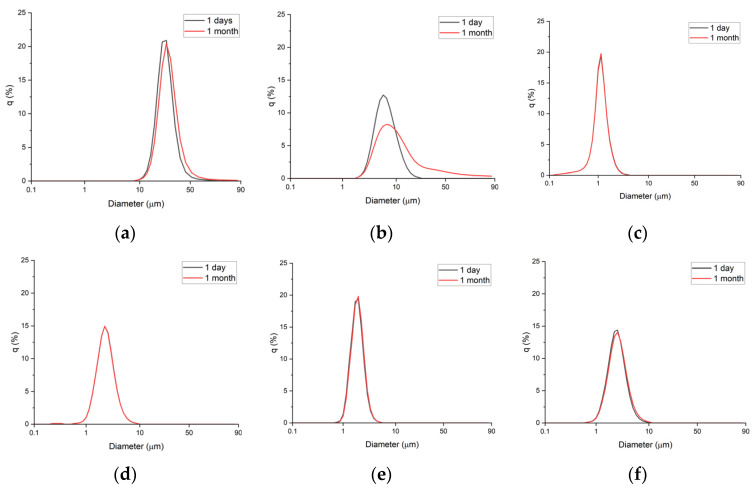
Particle size distributions after 1 day and after 1 month: (**a**) Xanthan gum (1.0 wt%), (**b**) Carbopol 981 (1.0 wt%), (**c**) TR-2 (0.5 wt%), (**d**) TR-2 (0.25 wt%), (**e**) Ultrez 20 (0.5 wt%), and (**f**) Ultrez (0.25 wt%).

**Figure 3:**
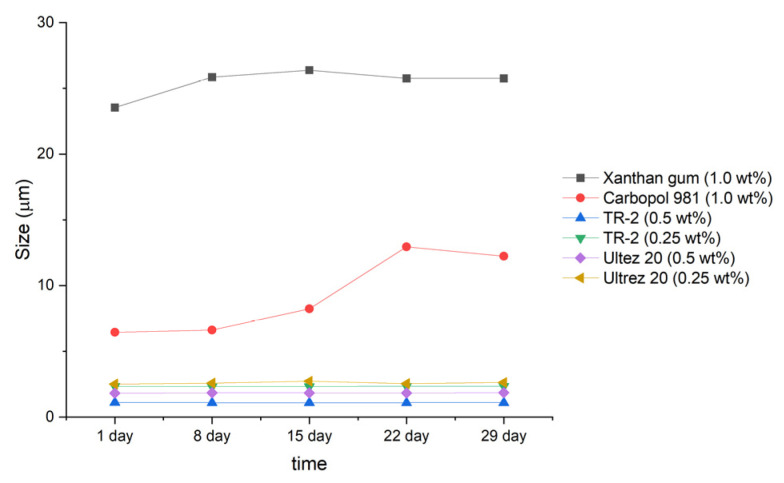
Particle size distribution of emulsions with respect to storage time.

Next, the emulsions were stored at 45 °C, and particle size measurements were carried out every week for 1 month (Figure 2 and Figure 3). The emulsions prepared with xanthan gum (1.0 wt%) and Carbopol 981 (1.0 wt%) showed large changes in particle size, growing to diameters of 2.2 and 6 µm, respectively.

In contrast, the four emulsions prepared with TR-2 and Ultrez 20 showed no changes in particle size during storage for 1 month at 45 °C. Therefore, highly stable emulsions can be prepared using small quantities of TR-2 and Ultrez 20.

#### 3.2.2. Emulsion Stability Determined Using Turbiscan Measurements

The high-temperature stability of the emulsions was measured using a Turbiscan device using backscattering data because of the high turbidity of the samples.

**Figure 4:**
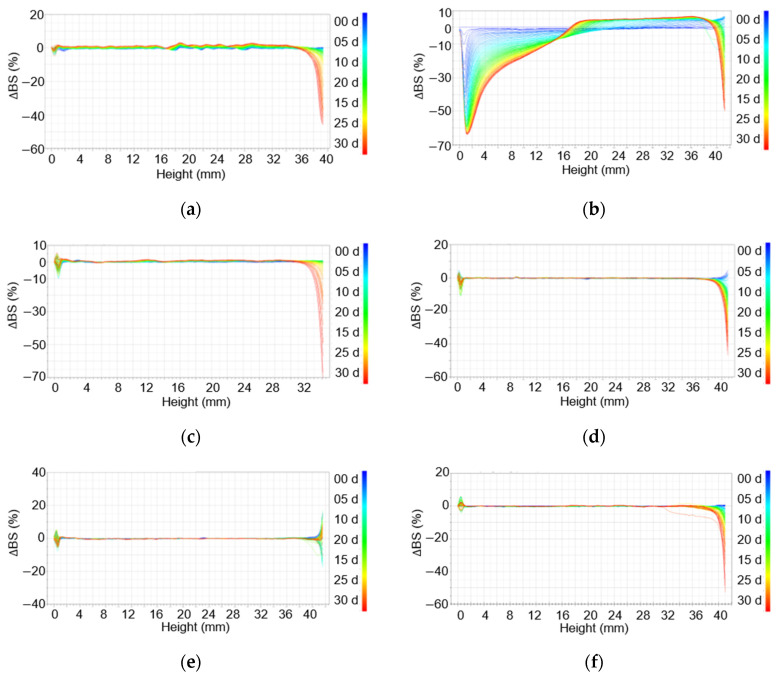
Delta backscattering of emulsions stored at 45 °C for a month: (**a**) Xanthan gum (1.0 wt%), (**b**) Carbopol 981 (1.0 wt%), (**c**) TR-2 (0.5 wt%), (**d**) TR-2 (0.25 wt%), (**e**) Ultrez 20 (0.5 wt%), and (**f**) Ultrez (0.25 wt%).

In the case of the emulsion prepared using xanthan gum (1.0 wt%), a large change in backscattering was observed over 30 days (Figure 4a). In addition, there were changes in scattering from the top to the bottom of the sample, suggesting phase separation. In particular, backscattering from the middle section increased, indicating oil aggregation or gelling agent entanglement. In addition, given that xanthan gum is a natural substance, the corresponding emulsion had grown mold by the fifth day after production (Figure S1 Supplementary Materials).

The emulsions prepared with Carbopol 981 (1.0 wt%) showed the largest change in backscattering among the six emulsions (Figure 4b). With an increase in time, backscattering from the middle to the top increased, and a creaming layer could be clearly observed. In addition, a decrease in delta backscattering values from the bottom to the middle section suggests a clarification of the liquid as a result of creaming. Further, in the top region, the delta backscattering value increases and then decreases, suggesting the close-packing or aggregation of the floating oil particles.

For the emulsions prepared with TR-2 (0.5 or 0.25 wt%) and Ultrez 20 (0.5 or 0.25 wt%), there was no significant change in stability over 1 month of storage at 45 °C (Figure 4c–f). However, in the samples containing 0.5 wt% of a gelling agent, the delta backscattering in the middle section was increased, confirming that the gelling agent was entangled in the emulsions. In contrast, for the emulsions prepared with 0.25 wt% of gelling agents (TR-2 and Ultrez 20), there was little change in delta backscattering, indicating that the use of a small amount of a gelling agent can yield highly stable emulsions.

**Figure 5:**
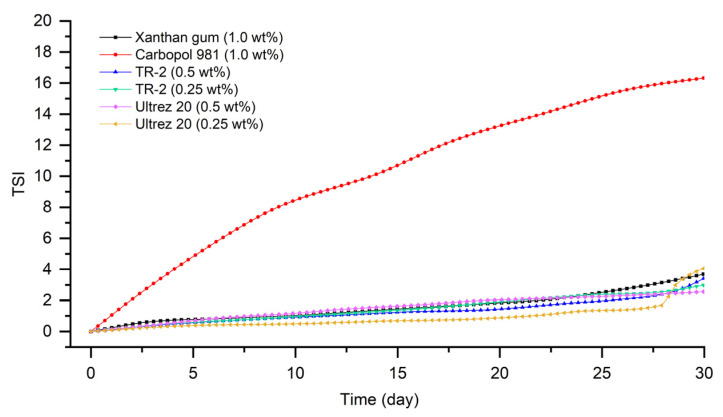
TSI values of emulsions stored at 45 °C for a month.

As shown in Figure 5, the emulsion prepared with Carbopol 981 was the most unstable, having the largest TSI value. The emulsion to which Ultrez 20 was added showed that the emulsion stability rapidly became unstable after 27 days from manufacturing. Next, it was confirmed that the stability index of the emulsion to which xanthan gum was added was low. The emulsion to which TR-2 (0.25 wt%) was added showed that the TSI value was calculated to be the smallest, indicating that it was the most stable state, as in delta backscattering data.

### 3.3. Stability under Harsh Conditions

**Figure 6:**
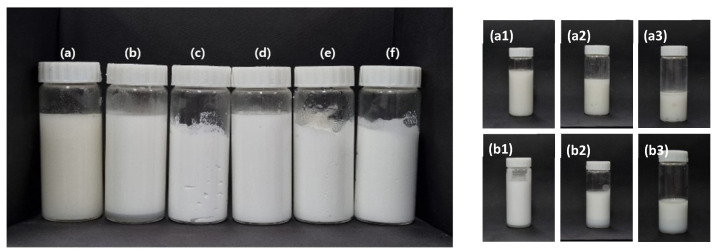
(Left) Emulsions prepared with gelling agents after 1 cycle of harsh treatment: (**a**) Xanthan gum (1.0 wt%), (**b**) Carbopol 981 (1.0 wt%). (**c**) TR-2 (0.5 wt%), (**d**) TR-2 (0.5 wt%), (**e**) Ultrez 20 (0.5 wt%), and (**f**) Ultrez 20 (0.25 wt%). (Right) Emulsions prepared with gelling agents in harsh environment: (**a1**–**a3**) Xanthan gum (1.0 wt%) and (**b1**–**b3**) Carbopol 981 (1.0 wt%) after 1, 3, and 5 cycles of harsh testing.

The six emulsion samples were exposed to temperature cycling, and photographs of the samples after one cycle are shown in the left-hand panel of Figure 6. The samples prepared with xanthan gum and Carbopol 981 showed the greatest changes during cycling under harsh conditions, as shown in the right-hand panel of Figure 6. After the first cycle, the emulsions containing xanthan gum and Carbopol 981 showed signs of phase separation. In particular, the xanthan gum emulsion showed a floating oil layer on the surface of the sample from the first cycle. After the third and fifth cycles, the floating oil layer separated, and aggregated oil was observed throughout the emulsion, as shown in Figure 6(a1–a3). Similarly, the emulsion prepared with Carbopol 981 was separated into an underlying water-gelling agent layer and emulsion-gelling agent layer after the first cycle, and the separation progressed in the third and fifth cycles, as shown in Figure 6(b1–b3). In the emulsions prepared using TR-2 and Ultrez 20, there was no change in the emulsion phase, independent of the amount of gelling agent added. Therefore, xanthan gum and Carbopol 981 are not suitable for stabilizing emulsions that will be subjected to harsh conditions. In contrast, TR-2 and Ultrez 20 greatly enhanced the emulsion stability, even at low contents.

## 4. Conclusions

In this study, gelling agents were tested for the preparation of stable, surfactant-free emulsions because such emulsions would have numerous applications, for example, in food, cosmetics, medicine, and ink. At room temperature, high temperatures, and under harsh conditions, the emulsions prepared with xanthan gum and Carbopol 981 showed low stability and suffered from phase separation and oil agglomeration. In particular, for the emulsions prepared with Carbopol 981, the emulsion phases showed significant changes at high temperatures and harsh conditions, yielding an oil that was not uniformly dispersed in the continuous phase (distilled water). The emulsion prepared with xanthan gum also lacked stability, suffering from oil separation at high temperatures and under harsh conditions.

In the case of the emulsions prepared using TR-2 and Ultrez 20, there was no change in the size of the oil particles at high temperatures and under harsh conditions, and the oil phase was maintained in a uniform state, without significant changes. Importantly, the emulsion should not have too high of a viscosity for uniform sampling, and the use of 0.5 wt% of TR-2 and Ultrez 20 was observed to result in extremely high viscosity, which is unsuitable for applications. In contrast, the use of 0.25 wt% TR-2 yielded a highly stable emulsion with uniform oil dispersion, even at high temperatures and under harsh conditions.

## Figures and Tables

**Table 1:** Emulsion preparation procedures.

Gelling Agent	Content [wt%]	Mixing 1	Mixing 2	Mixing 3	Defoaming
Xanthan gum	1.0	8000 rpm, 5 min with oil	8000 rpm, 20 min with gelling agent	8000 rpm, 20 min with neutralizer	180 s
Carbopol 981	1.0				
Pemulen TR-2	0.5, 0.25				
Carbopol Ultrez 20	0.5, 0.25	Soaking gelling agent 10 min	8000 rpm, 20 min with oil		

## Data Availability

Not applicable.

## Supplementary Materials

The following supporting information can be downloaded at: https://www.mdpi.com/article/10.3390/ma15186462/s1, Figure S1. Emulsion prepared with xanthan gum showing mold growth on the fifth day of storage.
