# Supplementary material for: A Comparison of Gelling Agents for Stable, Surfactant-Free Oil-in-Water Emulsions
Source: Materials (Basel). 2022 Sep 17;15(18):6462. doi: 10.3390/ma15186462 (PMC9502322; doi:10.3390/ma15186462)
Supplement: Supplementary file 1 [file materials-15-06462-s001.zip › materials-1898609-supplementary.pdf]

materials

MDPI

Supplementary Materials

A comparison of gelling agents for stable, surfactant-free oil-in-water emulsions

Ji Yun Lee[1,2], Sang Ho Lee[3], Seon Ae Hwangbo[1,*] and Tae Geol Lee[1,*]

1 Nano safety Team, Safety Measurement Institute, Korea Research Institute of Standards and Science (KRISS), 267 Gajeong-ro, Yuseong-gu, Daejeon 34113, Republic of Korea
2 Department of Chemical and Biomolecular Engineering, College of Engineering Yonsei University, 50 Yonsei-ro, Seodaemun-gu, Seoul 038722, Republic of Korea
3. LG Household & Health Care, Technical Process Research Team, 175 Gajeong-ro, Yuseong-gu, Daejeon, 34114, Republic Korea

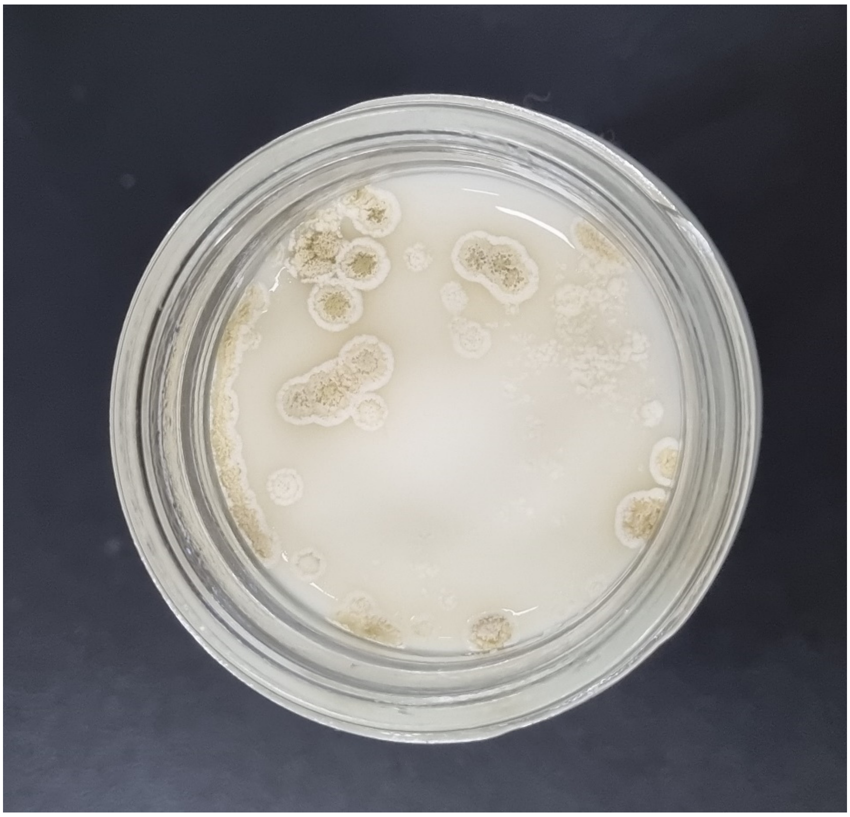

Figure S1. Emulsion prepared with xanthan gum showing mold growth on the fifth day of storage.
